# Human knee laxity in ACL-deficient and physiological contralateral joints: intra-operative measurements using a navigation system

**DOI:** 10.1186/1475-925X-13-86

**Published:** 2014-06-24

**Authors:** Pierre Imbert, Claudio Belvedere, Alberto Leardini

**Affiliations:** 1 Department of Knee Surgery, Clinique Notre Dame de la Merci, Saint-Raphaël, France; 2 Movement Analysis Laboratory, Centro di Ricerca Codivilla-Putti, Istituto Ortopedico Rizzoli, Via di Barbiano 1/10, 40136 Bologna, Italy

**Keywords:** Knee joint laxity, Knee instability tests, ACL-deficiency knee, Contralateral healthy knee, Knee surgical navigation, Knee biomechanics

## Abstract

**Background:**

The comprehension of human knee laxity and of the failures of relevant surgical reconstructions of the anterior cruciate ligament (ACL) can be enhanced by the knowledge of the laximetric status of the contralateral healthy knee (CHK). Rarely this is available in patients, directly from the skeletal structures, and for a number of the standard clinical tests. The general aim of this study was to measure the extent to which laxity occurs immediately before surgery in the ACL deficient knee (ADK) with respect to CHK, in a number of standard clinical evaluation tests.

**Method:**

Thirty-two patients with ACL deficiency were analyzed at ADK and at CHK by a navigation system immediately before reconstructions. Knee laxity was assessed based on digitized anatomical references during the antero-posterior drawer, Lachman, internal-external rotation, varus-valgus, and pivot-shift tests. Antero-posterior laxity was normalized based on patient-specific length of the tibial plateau.

**Results:**

In the drawer test, statistical significance (p < 0.05) was found for the larger antero-posterior laxity in ADK than in CHK, on average, of 54' in the medial and 47' in the lateral compartments, when measured in normalized translations. In the Lachman test, these were about 106' and 68'. The pivot-shift test revealed a significant 70' larger antero-posterior central laxity and a 32' larger rotational laxity. No statistically relevant differences were observed in the other tests.

**Conclusion:**

The first conclusion is that it is important to measure also the antero-posterior and rotational laxity of the uninjured contralateral knee in assessing the laxity of the injured knee. A second is that the Lachman test shows knee laxity better than the AP drawer, and that the pivot-shift test was the only one able to reveal rotational instability. The present original measurements and analyses contribute to the knowledge of knee joint mechanics, with possible relevant applications in biomedical and clinical research.

## Introduction

Current surgical treatments for antero-posterior laxity and rotational instability of the human knee, typically via reconstruction of the anterior cruciate ligament (ACL), allow a satisfactory correction and an acceptable subjective sensation of joint stability [1]. However, secondary degenerative changes occur as in non-surgically treated ACL lesions [2], these being due to inadequate restoration of physiological knee mobility and stability [3,4].

A number of tests are used to assess laxity and instability in biomechanical and clinical settings [1,5,6], such as the antero-posterior drawer, the Lachman, the varus-valgus stability, the internal-external rotation, and the pivot-shift. However, not all these tests are usually performed, and rarely skeletal differences among patients are considered [7]. Apparently, only the pivot-shift reproduces the combined rotational/translational instability in the ACL-deficient knees (ADK). In more details, the result of this test is positive when the lateral tibial plateau subluxates anteriorly with respect to the lateral femoral condyle while applying tibial internal rotation plus a valgus stress with the knee slightly flexed, although relevant grading relies on the examiner’s perception of instability that occurs during this manoeuvre [8].

The most recent surgical techniques for ACL reconstruction involve placing the ACL graft less vertically [9,10], or introducing an extra-articular reinforcement in addition to the intra-articular reconstruction [9] for a possibly better rotational control. However, non-physiological knee kinematics during activities have been reported also in patients after successful ACL reconstruction, i.e. with a negative pivot-shift examination [11,12]. Further investigations of perturbed knee laxity in ADK are, therefore, necessary to comprehend fully the biomechanical changes occurring after ACL injury. Particularly, in subjects with ADK the quantification of joint laxity also in the contralateral healthy knee (CHK) would offer a suitable reference to assess these alterations [6,13]. For a reliable such analysis, skeletal-based measurements should be taken *in-vivo* in both knees, but this has been reported only in a single study [14]. This work, however, was limited by the very small sample size, i.e. 5 patients, most of whom with the contralateral knee not fully normal.

Modern surgical navigation systems [15] for surgical ACL reconstructions, by tracking intra-operatively the anatomically-based six-degrees-of-freedom of the femur and tibia, can assist the surgeon in tunnel placement and in the assessment of joint kinematics and laxity in the ADK [16-18], but also in the CHK [18,19]. These navigation systems give access also to intra-operative additional measurements, potentially very relevant during ACL reconstruction surgery for quantification in laxity evaluation tests.

The general aim of this study was to determine the extent to which joint laxity is found in the ADK immediately before surgery, and, particularly, how this laxity compares with that at the CHK. More specific scopes were (a) the demonstration that the knowledge of antero-posterior and rotational laxity in the CHK is important for the comprehension of this laxity in the ADK, and (b) that there are tests able to reveal better the degree of laxity and instability in the ADK. For these scopes, for the first time knee joint motion was measured in a large cohort of 32 patients intra-operatively, i.e. immediately before ACL reconstruction, by using skeletal trackers and a navigation system. In this special condition, skeletal structures are accessible, for direct bone tracking and for patient-specific skeletal-based normalization. The full series of clinical tests used routinely to assess laxity were investigated. All these original measurements are here reported, separately for each knee.

## Patients and methods

Thirty-two ACL reconstructions were analyzed in as many patients (Table 1) within a year and a half timeframe by a single experienced surgeon. The inclusion criteria were: a) isolated ACL rupture, i.e. no varus-valgus laxity, meniscal lesion or cartilage damage evidenced by a questionnaire, physical examination and MRI, and b) uninjured contralateral knee, as assessed by a questionnaire and physical examination. A clinical assessment was also performed by using the International Knee Documentation Committee (IKDC) scoring system [20]. In the ADK, all patients had joint instability (IKDC score: C or D), with no clinical or radiological evidence of any other ligamentous lesion, degenerative change or meniscus lesion. In all patients the CHK was stable with no major ligament injuries or degenerative changes (IKDC score: A or B).

**Table 1:** Original values of APL (in millimeters) and ARR (in degrees) in both sides and directions, reported together with the corresponding AP-tib values used for normalization; these are reported for each patient analyzed and for all tests performed

**Patient**	**sex**	**age**	**AP-tib (mm)**		**DRAWER TEST**		**LACHMAN TEST**		**ROTATION TEST AT 20° FLEXION**					**ROTATION TEST AT 90° FLEXION**					**VAR-VAL TEST**		**PIVOT SHIFT TEST**	
					**Medial APL (mm)**	**Lateral APL (mm)**	**Medial APL (mm)**	**Lateral APL (mm)**	**Medial APL (mm)**	**Lateral APL (mm)**	**Total ARR (Deg)**	**External ARR (Deg)**	**Internal ARR (Deg)**	**Medial APL (mm)**	**Lateral APL (mm)**	**Total ARR (Deg)**	**External ARR (Deg)**	**Internal ARR (Deg)**	**Varus (Deg)**	**Valgus (Deg)**	**Central APL (mm)**	**Total ARR (Deg)**
**#1**	**m**	**32**	**53**	**CHK**	**5**	**13**	**3**	**10**	**20**	**18**	**24**	**12**	**12**	**22**	**26**	**32**	**16**	**16**	**1**	**2**	**7**	**17**
				**ADK**	**4**	**15**	**14**	**20**	**22**	**19**	**25**	**12**	**14**	**21**	**29**	**31**	**10**	**21**	**3**	**2**	**10**	**13**
**#2**	**m**	**27**	**55**	**CHK**	**1**	**7**	**4**	**7**	**11**	**16**	**19**	**9**	**10**	**13**	**23**	**27**	**15**	**12**	**1**	**1**	**5**	**12**
				**ADK**	**5**	**15**	**14**	**19**	**17**	**17**	**21**	**6**	**15**	**22**	**27**	**30**	**12**	**17**	**2**	**2**	**12**	**17**
**#3**	**m**	**37**	**53**	**CHK**	**2**	**8**	**4**	**11**	**17**	**21**	**28**	**17**	**11**	**21**	**28**	**35**	**14**	**22**	**2**	**1**	**7**	**14**
				**ADK**	**4**	**13**	**13**	**17**	**19**	**26**	**29**	**12**	**18**	**30**	**35**	**43**	**14**	**29**	**2**	**3**	**22**	**27**
**#4**	**m**	**23**	**54**	**CHK**	**4**	**7**	**5**	**12**	**16**	**21**	**25**	**14**	**11**	**23**	**27**	**37**	**26**	**11**	**1**	**1**	**8**	**16**
				**ADK**	**6**	**14**	**9**	**22**	**26**	**25**	**33**	**14**	**19**	**26**	**29**	**37**	**14**	**23**	**3**	**3**	**10**	**21**
**#5**	**m**	**29**	**52**	**CHK**	**4**	**9**	**5**	**14**	**12**	**17**	**19**	**9**	**10**	**20**	**29**	**34**	**19**	**15**	**1**	**2**	**5**	**17**
				**ADK**	**7**	**16**	**11**	**22**	**19**	**18**	**23**	**12**	**10**	**23**	**31**	**35**	**16**	**19**	**1**	**2**	**16**	**22**
**#6**	**m**	**20**	**52**	**CHK**	**5**	**12**	**8**	**13**	**13**	**15**	**16**	**9**	**8**	**20**	**23**	**29**	**10**	**18**	**2**	**1**	**8**	**16**
				**ADK**	**6**	**14**	**10**	**23**	**16**	**17**	**18**	**6**	**13**	**20**	**26**	**30**	**12**	**18**	**1**	**3**	**15**	**23**
**#7**	**m**	**21**	**60**	**CHK**	**4**	**7**	**5**	**12**	**19**	**23**	**28**	**18**	**11**	**23**	**29**	**35**	**21**	**15**	**1**	**1**	**10**	**15**
				**ADK**	**8**	**12**	**7**	**9**	**23**	**20**	**29**	**18**	**11**	**28**	**33**	**40**	**19**	**21**	**2**	**2**	**5**	**13**
**#8**	**m**	**23**	**60**	**CHK**	**3**	**9**	**7**	**11**	**15**	**13**	**18**	**10**	**9**	**22**	**25**	**32**	**14**	**17**	**2**	**2**	**7**	**15**
				**ADK**	**6**	**12**	**14**	**23**	**18**	**13**	**19**	**8**	**11**	**25**	**31**	**39**	**22**	**17**	**2**	**2**	**17**	**25**
**#9**	**m**	**20**	**52**	**CHK**	**5**	**8**	**4**	**12**	**14**	**16**	**23**	**17**	**6**	**23**	**30**	**40**	**21**	**19**	**1**	**1**	**6**	**11**
				**ADK**	**7**	**11**	**15**	**28**	**27**	**17**	**27**	**19**	**8**	**27**	**31**	**37**	**16**	**22**	**4**	**2**	**23**	**21**
**#10**	**m**	**39**	**65**	**CHK**	**4**	**10**	**7**	**14**	**15**	**19**	**22**	**10**	**12**	**19**	**27**	**31**	**11**	**19**	**2**	**1**	**7**	**14**
				**ADK**	**6**	**15**	**15**	**18**	**18**	**19**	**22**	**7**	**15**	**25**	**31**	**37**	**22**	**15**	**2**	**2**	**11**	**21**
**#11**	**m**	**28**	**49**	**CHK**	**4**	**7**	**3**	**9**	**12**	**19**	**23**	**16**	**7**	**23**	**35**	**42**	**20**	**22**	**1**	**1**	**7**	**12**
				**ADK**	**8**	**17**	**11**	**14**	**20**	**20**	**25**	**11**	**14**	**30**	**40**	**47**	**31**	**16**	**1**	**3**	**12**	**23**
**#12**	**m**	**37**	**54**	**CHK**	**4**	**9**	**6**	**9**	**15**	**19**	**23**	**11**	**13**	**18**	**25**	**31**	**23**	**8**	**0**	**2**	**11**	**19**
				**ADK**	**9**	**19**	**13**	**23**	**18**	**19**	**23**	**9**	**14**	**24**	**32**	**34**	**19**	**15**	**1**	**2**	**20**	**26**
**#13**	**f**	**46**	**35**	**CHK**	**3**	**3**	**15**	**13**	**21**	**20**	**25**	**19**	**6**	**27**	**33**	**43**	**19**	**24**	**4**	**2**	**8**	**16**
				**ADK**	**7**	**11**	**9**	**22**	**25**	**21**	**30**	**12**	**18**	**26**	**37**	**49**	**29**	**20**	**6**	**3**	**7**	**17**
**#14**	**m**	**25**	**49**	**CHK**	**7**	**7**	**12**	**14**	**38**	**29**	**37**	**3**	**34**	**41**	**39**	**45**	**11**	**34**	**3**	**3**	**3**	**24**
				**ADK**	**8**	**18**	**18**	**22**	**41**	**34**	**38**	**17**	**21**	**34**	**39**	**38**	**21**	**16**	**5**	**4**	**11**	**18**
**#15**	**m**	**32**	**36**	**CHK**	**5**	**10**	**9**	**19**	**16**	**21**	**23**	**12**	**11**	**19**	**27**	**31**	**23**	**9**	**3**	**3**	**8**	**11**
				**ADK**	**8**	**15**	**12**	**18**	**13**	**23**	**25**	**12**	**12**	**21**	**28**	**35**	**14**	**21**	**2**	**3**	**12**	**20**
**#16**	**f**	**19**	**36**	**CHK**	**6**	**13**	**7**	**10**	**26**	**27**	**41**	**20**	**20**	**30**	**37**	**52**	**19**	**33**	**1**	**2**	**9**	**22**
				**ADK**	**5**	**22**	**15**	**22**	**22**	**27**	**38**	**23**	**15**	**27**	**29**	**43**	**25**	**7**	**3**	**2**	**18**	**26**
**#17**	**m**	**33**	**55**	**CHK**	**5**	**13**	**6**	**15**	**18**	**17**	**22**	**11**	**10**	**28**	**34**	**39**	**21**	**19**	**1**	**2**	**6**	**18**
				**ADK**	**8**	**17**	**12**	**18**	**26**	**16**	**24**	**15**	**9**	**29**	**33**	**39**	**20**	**20**	**3**	**3**	**10**	**15**
**#18**	**m**	**33**	**42**	**CHK**	**2**	**9**	**5**	**12**	**10**	**16**	**18**	**12**	**6**	**15**	**29**	**29**	**12**	**17**	**3**	**2**	**8**	**18**
				**ADK**	**7**	**15**	**18**	**25**	**21**	**18**	**25**	**17**	**8**	**24**	**33**	**35**	**19**	**16**	**2**	**2**	**26**	**18**
**#19**	**m**	**16**	**44**	**CHK**	**8**	**13**	**6**	**13**	**10**	**14**	**15**	**10**	**6**	**22**	**30**	**37**	**27**	**10**	**1**	**2**	**10**	**18**
				**ADK**	**5**	**15**	**10**	**20**	**19**	**16**	**21**	**8**	**13**	**28**	**31**	**38**	**12**	**25**	**2**	**2**	**15**	**24**
**#20**	**m**	**22**	**53**	**CHK**	**7**	**16**	**4**	**13**	**16**	**14**	**16**	**10**	**6**	**25**	**30**	**33**	**18**	**15**	**0**	**1**	**14**	**12**
				**ADK**	**5**	**16**	**14**	**23**	**22**	**25**	**24**	**13**	**11**	**25**	**32**	**33**	**9**	**24**	**1**	**2**	**13**	**20**
**#21**	**f**	**36**	**46**	**CHK**	**5**	**11**	**3**	**15**	**13**	**24**	**30**	**17**	**13**	**17**	**30**	**42**	**28**	**13**	**2**	**2**	**18**	**17**
				**ADK**	**7**	**12**	**12**	**29**	**25**	**24**	**34**	**25**	**9**	**24**	**33**	**43**	**20**	**23**	**2**	**3**	**24**	**28**
**#22**	**m**	**46**	**56**	**CHK**	**5**	**12**	**7**	**7**	**19**	**25**	**25**	**10**	**15**	**25**	**28**	**33**	**20**	**14**	**1**	**1**	**11**	**15**
				**ADK**	**9**	**12**	**14**	**27**	**22**	**22**	**25**	**14**	**11**	**23**	**28**	**29**	**12**	**18**	**1**	**2**	**26**	**20**
**#23**	**m**	**20**	**60**	**CHK**	**4**	**9**	**5**	**10**	**17**	**21**	**27**	**11**	**16**	**24**	**36**	**45**	**25**	**20**	**1**	**1**	**9**	**19**
				**ADK**	**6**	**14**	**15**	**24**	**29**	**23**	**35**	**11**	**24**	**30**	**35**	**45**	**27**	**18**	**3**	**2**	**18**	**28**
**#24**	**m**	**48**	**56**	**CHK**	**3**	**13**	**6**	**18**	**19**	**19**	**22**	**8**	**14**	**19**	**28**	**32**	**19**	**13**	**1**	**1**	**9**	**14**
				**ADK**	**5**	**14**	**12**	**25**	**22**	**20**	**24**	**13**	**11**	**20**	**28**	**29**	**16**	**13**	**2**	**2**	**12**	**16**
**#25**	**m**	**19**	**53**	**CHK**	**4**	**8**	**6**	**13**	**15**	**16**	**23**	**10**	**12**	**20**	**25**	**33**	**11**	**21**	**1**	**1**	**7**	**19**
				**ADK**	**7**	**16**	**11**	**24**	**26**	**20**	**29**	**15**	**14**	**31**	**36**	**45**	**28**	**17**	**2**	**2**	**11**	**22**
**#26**	**m**	**21**	**58**	**CHK**	**5**	**8**	**11**	**21**	**24**	**23**	**32**	**14**	**18**	**32**	**36**	**49**	**31**	**18**	**1**	**1**	**9**	**17**
				**ADK**	**10**	**17**	**14**	**19**	**20**	**27**	**28**	**20**	**8**	**29**	**33**	**42**	**3**	**38**	**1**	**2**	**16**	**25**
**#27**	**m**	**38**	**49**	**CHK**	**3**	**12**	**3**	**12**	**11**	**17**	**21**	**11**	**10**	**19**	**32**	**39**	**19**	**20**	**2**	**1**	**10**	**15**
				**ADK**	**5**	**16**	**12**	**20**	**18**	**20**	**26**	**15**	**11**	**25**	**35**	**41**	**21**	**20**	**1**	**2**	**14**	**17**
**#28**	**m**	**23**	**58**	**CHK**	**5**	**14**	**3**	**16**	**17**	**22**	**27**	**17**	**10**	**25**	**32**	**43**	**26**	**17**	**1**	**1**	**16**	**20**
				**ADK**	**7**	**14**	**8**	**23**	**27**	**20**	**30**	**13**	**17**	**31**	**34**	**43**	**19**	**23**	**1**	**2**	**10**	**22**
**#29**	**m**	**36**	**49**	**CHK**	**6**	**21**	**8**	**17**	**20**	**21**	**28**	**17**	**11**	**32**	**39**	**49**	**21**	**28**	**1**	**1**	**7**	**15**
				**ADK**	**11**	**16**	**19**	**27**	**29**	**20**	**30**	**18**	**12**	**34**	**38**	**49**	**26**	**23**	**1**	**3**	**14**	**25**
**#30**	**m**	**46**	**48**	**CHK**	**2**	**15**	**8**	**12**	**16**	**14**	**21**	**13**	**9**	**26**	**35**	**45**	**17**	**28**	**2**	**1**	**10**	**16**
				**ADK**	**7**	**20**	**17**	**22**	**23**	**15**	**24**	**9**	**15**	**32**	**38**	**52**	**31**	**22**	**2**	**2**	**20**	**21**
**#31**	**m**	**35**	**49**	**CHK**	**5**	**11**	**3**	**9**	**11**	**13**	**18**	**15**	**4**	**23**	**31**	**40**	**25**	**15**	**1**	**2**	**15**	**26**
				**ADK**	**6**	**17**	**13**	**26**	**19**	**10**	**14**	**9**	**5**	**25**	**32**	**39**	**19**	**20**	**2**	**2**	**20**	**32**
**#32**	**m**	**19**	**53**	**CHK**	**3**	**7**	**3**	**16**	**13**	**23**	**25**	**12**	**13**	**17**	**31**	**37**	**23**	**14**	**1**	**1**	**6**	**11**
				**ADK**	**5**	**13**	**9**	**15**	**20**	**23**	**28**	**12**	**16**	**25**	**37**	**42**	**19**	**23**	**2**	**3**	**8**	**21**

All patients were asked before surgery to allow intra-operative data collection from both the ADK and CHK, according to an established technique [14] suitably adapted, and provided written informed consent as approved by the local Ethics Committee. No surgical complications occurred. No persistent pain was reported then by the patients.

**Figure 1:**
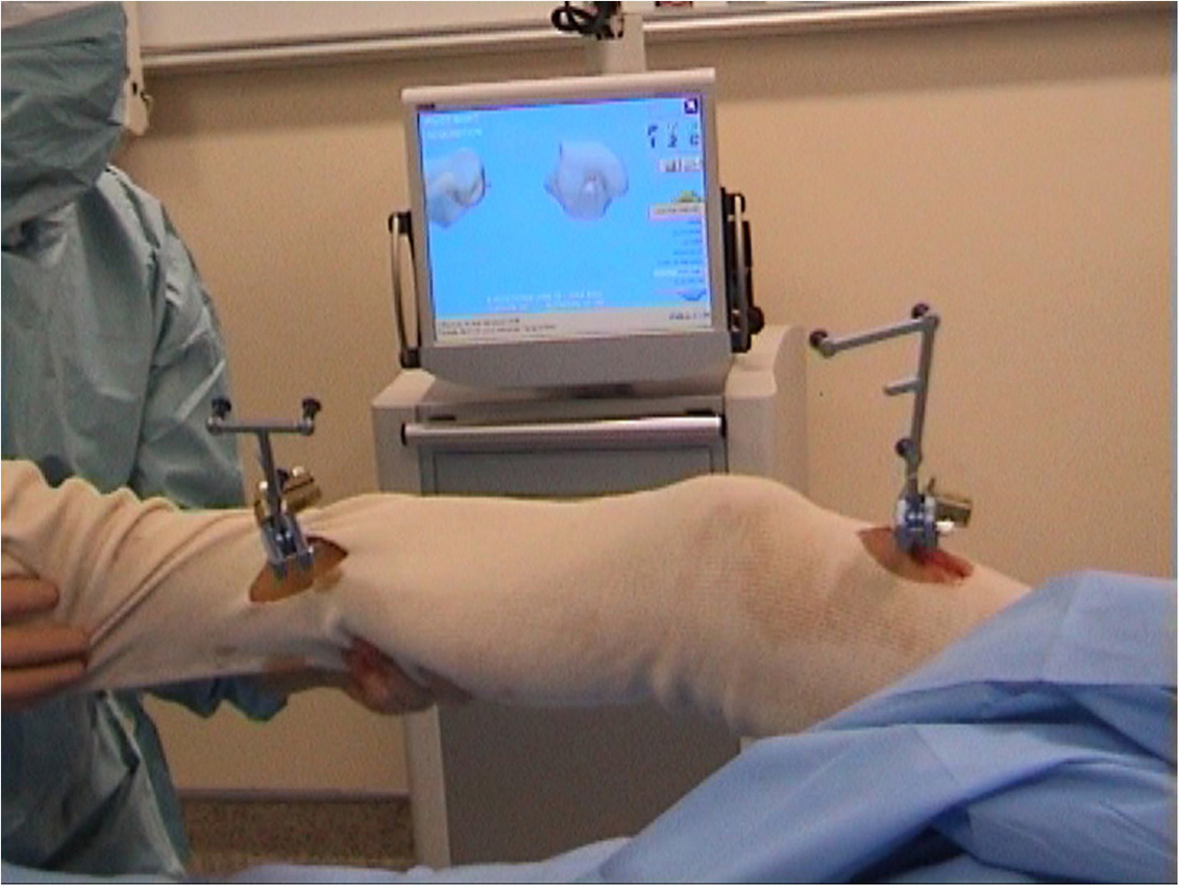
**Measurement set-up while performing the tests in surgery.** Bone pins are fixed onto the femur/tibia; cluster compounds of three passive reflecting markers are mounted onto these pins; both clusters communicate with the localizer (not visible); relevant data are shown on the monitor.

During surgery, an image-free passive-optical surgical navigation system for ACL reconstruction (Praxim, La Tronche, France; Figure 1) was used, but only for the necessary intra-operative measurements, i.e. not to assist surgery. For these systems and interventions the accuracy reported is 1° and 1 mm [18,21]. The system provides in real-time knee flexion-extension, varus-valgus, and internal-external rotation, i.e. the axial rotation, by means of bone trackers implanted in the femur and tibia [18,21]. Relevant bone tracking accuracy is within 1 mm and 1°, as reported in a previous paper testing the same navigation system using robotic machines [19]. The system consists of three marker clusters, used for bone tracking, and a workstation, equipped with a processing unit with dedicated software, a monitor, and a localizer, functioning as infrared light emitter and receiver. A reference frame is embedded in the localizer and in all clusters. A pointer-like cluster was used to digitize the anatomical landmarks necessary to define the patient-specific geometrical model of the knee and its reference frames.

**Figure 2:**
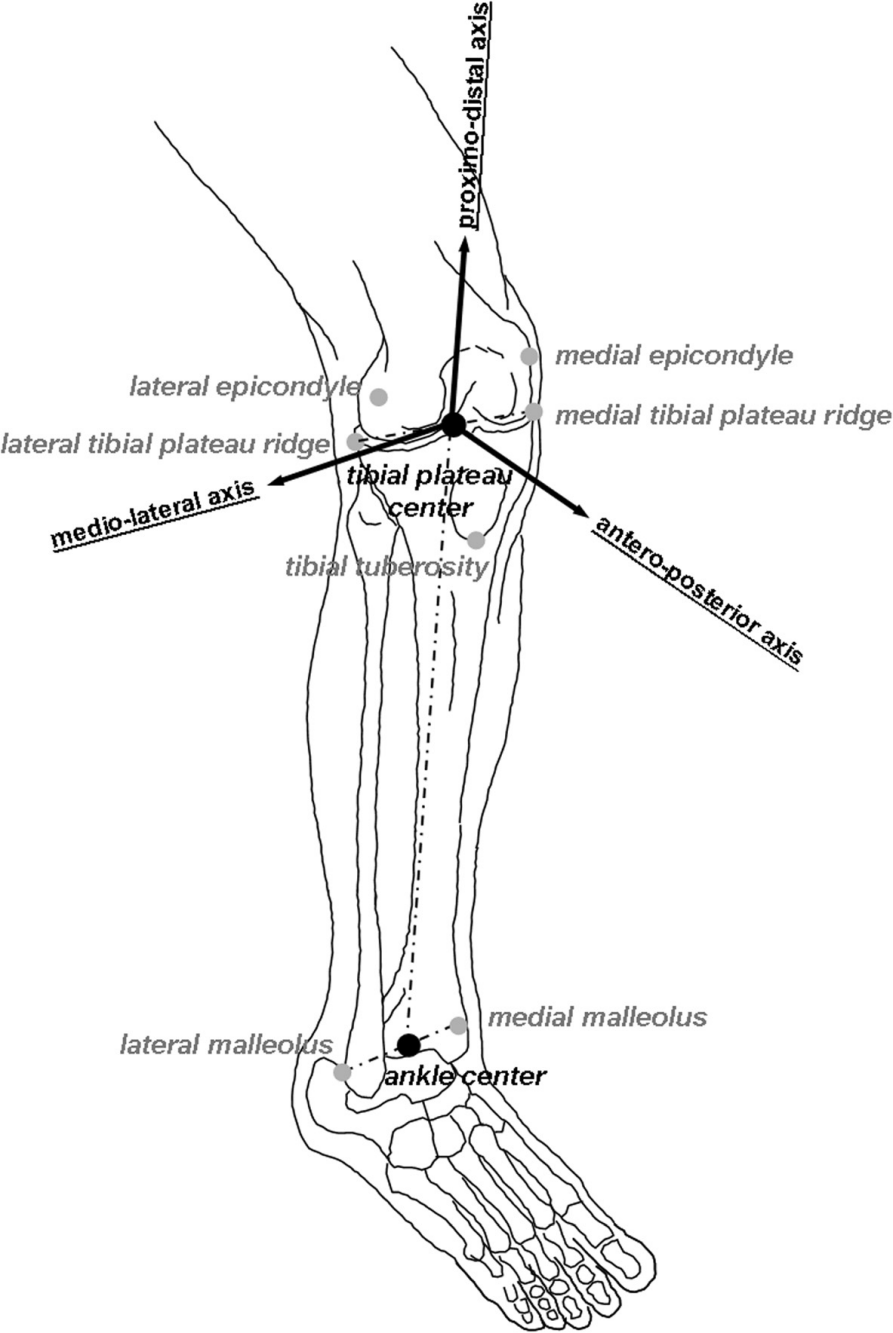
**Diagram with the anatomical landmarks used for defining the reference frames, those digitized directly (in grey) and those calculated as mid points (black).** The tibial anatomical reference frame with its three axes is also depicted.

The CHK was analyzed first. Steinmann pins were inserted into the distal femur and proximal tibia, corresponding clusters were mounted, the navigation system was initialized, and the pose of the knee in full extension and in neutral internal-external rotation according to surgeon’s examination was recorded. The following anatomical landmarks were digitized percutaneously by the pointer after careful external palpation (Figure 2): the medial and lateral epicondyles, the medial and lateral malleoli, the most prominent part of the tibial tuberosity, and the most medial and lateral points of the ridge respectively of the tibial plateau. The tibial anatomical reference frame was defined with the origin in the midpoint between the latter landmarks, a proximo-distal axis as the line passing through the origin and the ankle center, i.e. the midpoint between the two malleoli, and a mid-sagittal plane was defined as the mean plane, on a least-square approach, of the trajectories of the origin and of the ankle center during an imposed cycle of knee flexion and extension [21]. The medio-lateral axis was the orthogonal to the sagittal plane, the antero-posterior axis the orthogonal to the other two axes [21]. The femur anatomical reference frame was made to coincide with that of the tibia in knee full extension, and then tracked by the femoral cluster; the origin was defined in the midpoint between the two epicondyles [21]. The following tests were then performed [5] on CHK: the antero-posterior (AP) drawer, the Lachman, the internal-external rotation at 20° and 90° knee flexion, the varus-valgus stability at knee full extension, and the pivot-shift. Then, the clusters were moved from the CHK to the ADK, and the same procedures replicated. Navigation instruments were then removed and the ACL reconstruction was performed using the traditional, i.e. not navigated, surgical procedure.

The antero-posterior laxity (APL) was defined as the range, expressed in mm, of antero-posterior translation of a point of the femur in the tibial transverse plane. The axial rotation range (ARR), expressed in degrees, was also defined. During test executions in the CHK and the ADK, these parameters were stored from the navigation system (Table 1). In particular: 1) the APL in medial and lateral compartments, respectively of the medial and lateral epicondyles, obtained both during the drawer and the Lachman tests, and also during the internal-external rotation test at both 20° and 90° knee flexion; 2) the total ARR and corresponding internal and external sub-ranges obtained during the rotation test at 20° and 90° knee flexion; 3) the range of varus and valgus motion obtained during the varus-valgus stability test; 4) the total ARR and the central APL, i.e. of the origin of the femoral reference frame, obtained during the pivot-shift test.

From the digitized landmarks the antero-posterior tibial dimension (AP-tib) was calculated for each knee, based on the distance between the centre of the tibial plateau and the digitized tibial tuberosity once projected on the tibial transverse plane, and on a length-to-width ratio taken in a previous anatomical study [7]. To consider possible differences associated to skeletal size variations, APL data were then normalized, i.e. reported in percentage of the corresponding AP-tib ('AP-tib).

Data were analyzed in terms of mean values and standard deviations. All possible differences between CHK and ADK, between tests, and between compartments, were sought by t-test or paired t-test where appropriate. Furthermore, the Pearson product–moment correlation coefficient (R) was also used to derive correlations between variables, and here reported in its squared form (R^2^), i.e. the coefficient of determination. Corresponding p-values were reported for assessing significance, this being accepted at p < 0.05. The patient population size analyzed in this study meets the criteria for achieving all these differences with 80' statistical power and an α-level of 0.05. All calculations were made in MatLab® software package (The MathWorks, Inc., Natick, MA-USA).

## Results

General demographic statistical analysis showed, as expected, a smaller antero-posterior tibial dimension in females than in males (R^2^ = 0.328, p = 0.001). In females, also total (R^2^ = 0.213, p = 0.008) and external (R^2^ = 0.270, p = 0.002) ARR, and corresponding lateral APL (R^2^ = 0.131, p = 0.042), were larger. Furthermore, a significant inverse correlation, though moderate (R = 0.585), was observed between the antero-posterior tibial dimension and the range of varus-valgus (R^2^ = 0.342, p < 0.001).

Here below, for each of the clinical tests performed and also for couples of tests, intra-subjects comparisons are first reported, i.e. ADK versus CHK; then inter-tests comparisons, and medial versus lateral compartment and internal versus external rotation comparisons are also discussed.

### AP drawer and lachman tests

**Figure 3:**
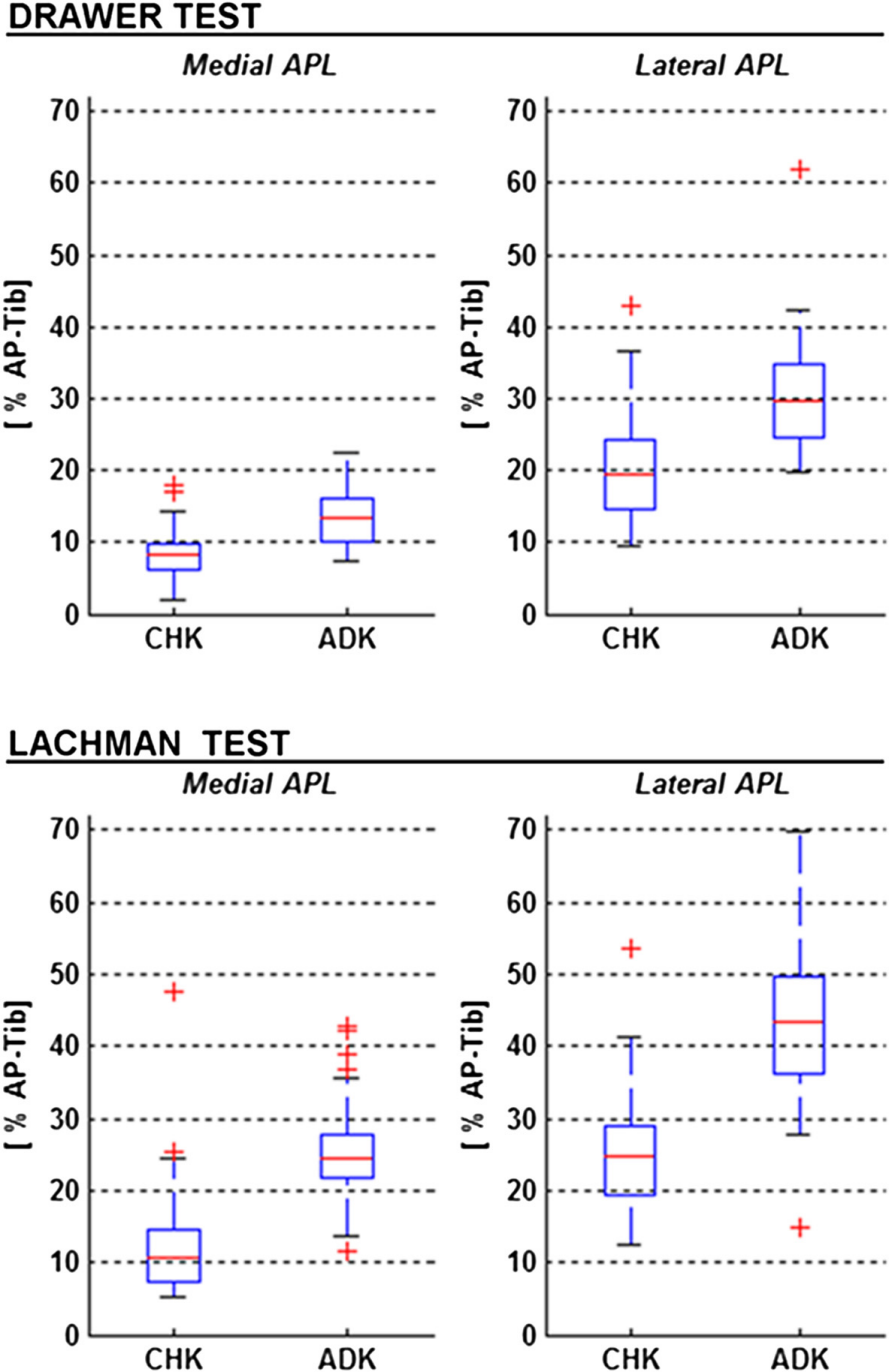
**Box-plots from the Drawer (top) and Lachman (bottom) tests.** The range of APL is shown for the medial (left) and lateral (right) compartments, both for the CHK and ADK. In each plot, the boxes have lines at the lower, median, and upper quartile values over the whole patient cohort; the whisker lines extending from each end of the box show the extent of the rest of the data; values for any outliers are reported beyond the ends of the whiskers.

Significantly larger APL was found in ADK than in CHK (Figure 3; Table 2). Particularly, in the AP drawer, this was, on average, of about 54' in the medial and 47' in the lateral compartments, the mean over the two compartments being 51'. In the Lachman test, these were much larger, i.e. 106', 68' and 87', respectively.

**Table 2:** Mean values of APL, expressed as percentage of AP-tib, reported for the two tests, and for the medial and lateral compartments of both knees.

			**CHK**		**ADK**		***R***^***2***^_***CHK→ADK;***_ ***; p***_***CHK→ADK***_
			**Mean ± SD**	***R***^***2***^_***med→lat;***_ ***; p***_***med→lat***_	**Mean ± SD**	***R***^***2***^_***med→lat;***_ ***; p***_***med→lat***_	
**APL [' AP-tib]**	**AP drawer**						
		**medial**	**8.7 ± 3.7**	***R***^***2***^ ***= 0.495; p < 0.001***	**13.4 ± 4.0**	***R***^***2***^ ***= 0.635; p < 0.001***	***R***^***2***^ ***= 0.273; p < 0.001***
		**lateral**	**20.6 ± 7.8**		**30.3 ± 8.3**		***R***^***2***^ ***= 0.275; p < 0.001***
	**Lachman**						
		**medial**	**12.4 ± 8.3**	***R***^***2***^ ***= 0.395; p < 0.001***	**25.6 ± 7.4**	***R***^***2***^ ***= 0.458; p < 0.001***	***R***^***2***^ ***= 0.423; p < 0.001***
		**lateral**	**25.7 ± 8.4**		**43.2 ± 11.6**		***R***^***2***^ ***= 0.439; p < 0.001***
	***R***^***2***^_***AP drawer→Lachman***_ ***; p***_***AP drawer→Lachman***_						
		***medial***	***R***^***2***^ ***= 0.100; p = 0.025***		***R***^***2***^ ***= 0.521; p < 0.001***		
		***lateral***	***R***^***2***^ ***= 0.100; p = 0.015***		***R***^***2***^ ***= 0.298; p < 0.001***		

For all possible couples of these values, R^2^ and p are also reported, in Italic.

By comparing these two tests and also looking separately at the two knees, significantly larger APL was found in Lachman test than in AP drawer. Particularly, this was, on average, of about 43' in the medial and 25' in the lateral compartments in the CHK, the mean over the two compartments being 34'; these values in the ADK were respectively 91', 43' and 67'.

Significantly larger APL was found in the lateral than in the medial compartment; the percentage difference for the CHK was, on average, 137' in the AP drawer test and 107' in the Lachman test, whereas for the ADK these were 126' and 69'.

### Internal-external rotation tests at 20° and 90° knee flexion

**Figure 4:**
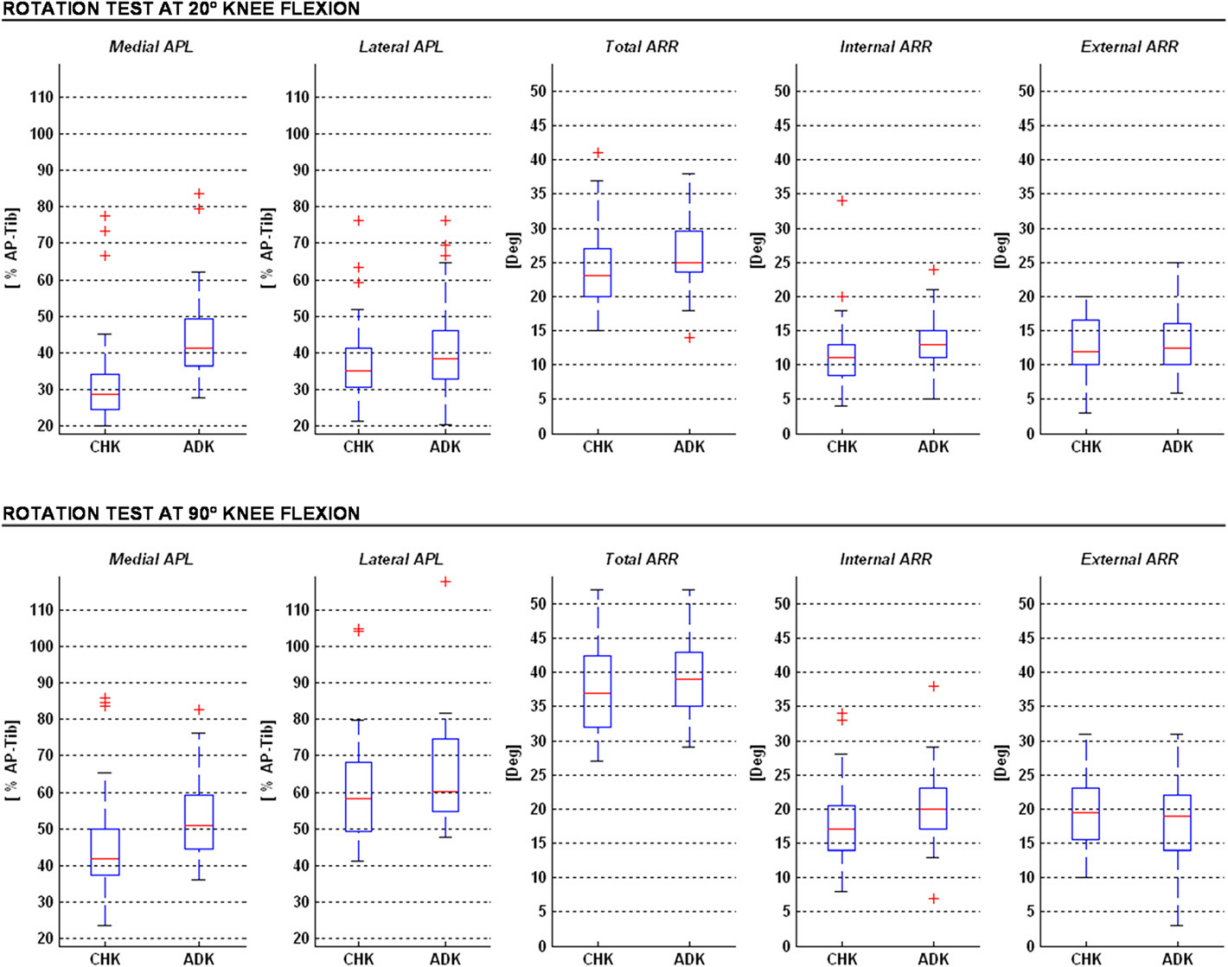
**Box-plots (same graphical representation as in Figure**4**) from the two rotation tests, at 20° (top) and 90° (bottom) knee flexions.** Ranges of medial and lateral APL, and total, internal and external ARR are reported both for the unaffected (CHK) and affected (ADK) knees.

In both tests no relevant differences were observed in laxity between the CHK and the ADK (Figure 4; Table 3) in terms of ARR values and the lateral APL; only in the test at 20° a significantly larger medial APL was observed in ADK than in CHK, this being of about 9'.

**Table 3:** Mean values of ARR, expressed in degrees, and APL, expressed as percentage of AP-tib, for the Internal-External rotation tests; these are reported for the medial and lateral compartments of both knees, and for the two joint positions.

			**CHK**		**ADK**		***R***^***2***^_***CHK→ADK;***_ ***; p***_***CHK→ADK***_
			**Mean ± SD**	***R***^***2***^_***intra→extra;***_ ***; p***_***intra→extra***_	**Mean ± SD**	***R***^***2***^_***intra→extra; ***_ ***;p***_***intra→extra***_	
**ARR [deg]**	**20° Knee Flexion**						
		**intra**	**11.4 ± 5.5**	***R***^***2***^ ***= 0.012; p = 0.293***	**13.2 ± 4.0**	***R***^***2***^ ***= 0.001; p = 0.932***	***R***^***2***^ ***= 0.035; p = 0.139***
		**extra**	**12.6 ± 3.8**		**13.3 ± 4.6**		***R***^***2***^ ***= 0.006; p = 0.538***
		**tot**	**23.9 ± 5.8**		**26.4 ± 5.5**		***R***^***2***^ ***= 0.051; p = 0.073***
	**90° Knee Flexion**						
		**intra**	**18.0 ± 6.3**	***R***^***2***^ ***= 0.017; p = 0.301***	**20.0 ± 5.3**	***R***^***2***^ ***= 0.013; p = 0.374***	***R***^***2***^ ***= 0.030; p = 0.172***
		**extra**	**19.5 ± 5.4**		**18.7 ± 6.7**		***R***^***2***^ ***= 0.005; p = 0.567***
		**tot**	**37.5 ± 6.5**		**39.0 ± 6.1**		***R***^***2***^ ***= 0.014; p = 0.345***
	***R***^***2***^_***20°→90°***_ ***; p***_***20°→90°***_						
		***intra***	***R***^***2***^ ***= 0.246; p < 0.001***		***R***^***2***^ ***= 0.350; p < 0.001***		
		***extra***	***R***^***2***^ ***= 0.359; p < 0.001***		***R***^***2***^ ***= 0.184; p = 0.001***		
		***tot***	***R***^***2***^ ***= 0.560; p < 0.001***		***R***^***2***^ ***= 0.551; p < 0.001***		
			**CHK**		**ADK**		***R***^***2***^_***CHK→ADK;***_ ***; p***_***CHK→ADK***_
			**Mean ± SD**	***R***^***2***^_***med→lat;***_ ***; p***_***med→lat***_	**Mean ± SD**	***R***^***2***^_***med→lat;***_ ***; p***_***med→lat***_	
**APL [' AP-tib]**	**20° Knee Flexion**						
		**medial**	**33.3 ± 14.2**	***R***^***2***^ ***= 0.039; p = 0.117***	**44.1 ± 12.8**	***R***^***2***^ ***= 0.017; p = 0.311***	***R***^***2***^ ***= 0.150; p << 0.001***
		**lateral**	**38.5 ± 12.0**		**41.1 ± 13.0**		***R***^***2***^ ***= 0.011; p = 0.407***
	**90° Knee Flexion**						
		**medial**	**46.0 ± 15.1**	***R***^***2***^ ***= 0.194; p < 0.001***	**52.7 ± 11.4**	***R***^***2***^ ***= 0.197; p < 0.001***	***R***^***2***^ ***= 0.060; p = 0.052***
		**lateral**	**60.9 ± 15.8**		**65.4 ± 14.4**		***R***^***2***^ ***= 0.022; p = 0.246***
	***R***^***2***^_***20°→90°***_ ***; p***_***20°→90°***_						
		***medial***	***R***^***2***^ ***= 0.163; p < 0.001***		***R***^***2***^ ***= 0.101; p = 0.008***		
		***lateral***	***R***^***2***^ ***= 0.398; p < 0.001***		***R***^***2***^ ***= 0.446; p < 0.001***		

For all possible couples of these values, R^2^ and p are also reported, in Italic.

By comparing these two tests, significantly larger values for the medial and lateral APL, and total and corresponding internal and external ARR were found at 90° flexion than at 20° flexion. Particularly, in the CHK these five values were of about 38', 58', 57', 58' and 55' respectively. This was observed also in the ADK, corresponding values being 20', 59', 48', 52' and 41'.

The lateral APL was significantly larger than the medial APL only in the rotation test at 90° flexion, this being of about 32' in the CHK and 24' in the ADK.

Neither in the CHK nor in the ADK were significant differences observed between internal and external ARR.

### Varus-valgus stability test

**Figure 5:**
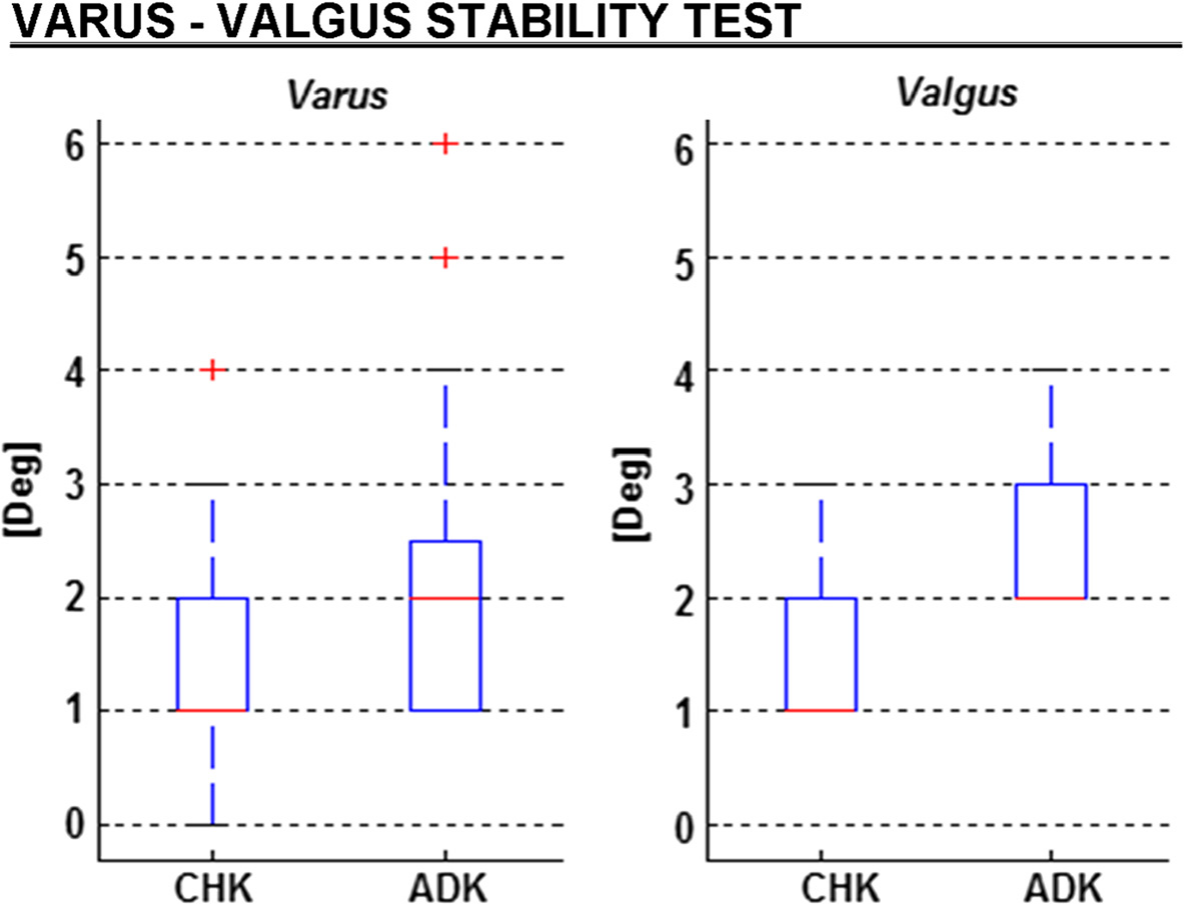
**Box-plots (same graphical representation as in Figure**3**) from the varus-valgus stability test.** Ranges of varus and valgus knee rotations are reported for both for the unaffected (CHK) and affected (ADK) knees.

This test revealed that the ADK was only slightly more unstable than the CHK. In CHK, the mean varus and valgus ranges were respectively 1.4° and 1.5° (Figure 5). In ADK, these were 2.1° and 2.4°. Particularly, significantly larger values were found in ADK than in CHK of about 50' (R^2^ = 0.101; p = 0.011) and 70' (R^2^ = 0.380; p < 0.001), respectively. In both knees, no significant difference (p = 0.870) was observed between the varus and the valgus ranges.

### Pivot-shift test

**Figure 6:**
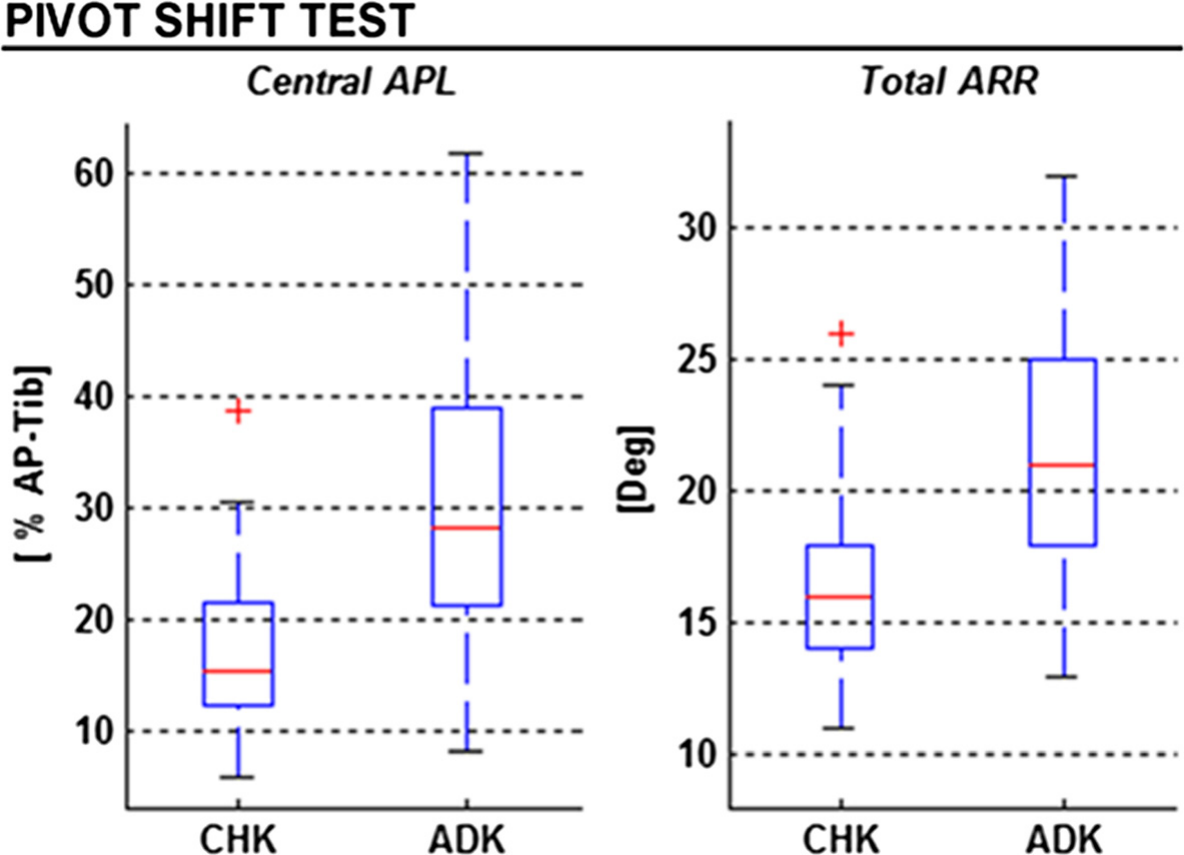
**Box-plots (same graphical representation as in Figure**3**) from the pivot-shift test.** Ranges of central APL and total ARR are reported for both for the unaffected (CHK) and affected (ADK) knees.

In the CHK, the mean range of the central APL was 17.6' AP-tib, the total ARR was 16.3° (Figure 6). In the ADK, this test was positive in all patients, and these values were, respectively, 29.9' AP-tib and 21.5°. Particularly, the values in the ADK were significantly larger than in CHK, these being of about 70' (R^2^ = 0.280; p < 0.001), and 32' (R^2^ = 0.297; p < 0.001), respectively.

## Discussion

The first finding for the present study (scope a) was that it is important to measure the antero-posterior and rotational laxity of the uninjured contralateral knee in assessing the laxity of the injured knee. Both knees are routinely examined by orthopaedic surgeons when assessing patients with knee injury, but this is a qualitative procedure. Our conclusion is corroborated by a quantitative comparative analysis of reliable skeletal measurements obtained in a large number of operations and also with dimensional normalization. A large part of the laxity in ADK can be due to that in the CHK: about two/thirds in the AP drawer, about half in the Lachman (Table 2). The importance of the comparison between CHK and ADK is therefore highlighted, thus supporting further the recommendation that the assessment of knee instability and laxity should be performed first within the same subject. The present ADK-to-CHK difference contributed also to reducing considerably the inter-patient variability.

A second finding (scope b) was that the pivot-shift was the only test able to reveal a significantly different rotational instability. The results from the present intra-operative measurements also confirmed that the expected higher translational instability in the ADK compared to that in the CHK may vary between the six tests performed, and that APL is associated to a higher rotational instability only in the pivot-shift. This test, therefore, must be considered the most suitable for revealing these differences, as previously suggested [18,22]. For all tests, the antero-posterior laxity in CHK was higher in the lateral than in the medial compartment, and this was more marked in the ADK.

In addition, by considering only mean values over the two compartments, in the ADK the APL was about 67' (mean between 91' and 43') larger in the Lachman test than in the AP drawer. With the knowledge of the CHK, the ADK to CHK differences can be calculated: APL was here found larger in ADK by 51' in the AP drawer and 87' in the Lachman test, i.e. 73' larger in the Lachman than in the AP drawer. In other words, Lachman is more revealing than AP drawer with the knowledge of CHK (73') rather than the ADK alone (67'), i.e. a 6' difference.

To our knowledge, only one previous study has performed similar joint laxity measures *in-vivo*, directly in skeletal structures, and with the comparison between affected and unaffected knees [14]. In that study, only five subjects were assessed (two of which with an unstable meniscus lesion in the unaffected knee) during the antero-posterior drawer and the internal-external rotation test at every 15° knee flexion step, between 15° and 90°. The present results compare well with those reported in another *in-vivo* study [23], though knees treated for a meniscal tear or with acute ACL injury were analyzed there. Knee laxity data were reported also from *in-vitro* studies, but the mechanical response to dynamic loading in specimens can be highly affected by altered viscoelastic properties [24]. In addition, ACL lesions are difficult to simulate *in-vitro*, because the typical complex conditions which occur after traumatic ruptures cannot be reproduced [25].

The present study is not free from limitations. Although the surgical navigation system was shown to be accurate enough for single point digitization [19], inaccuracies in anatomical landmark identification might affect relevant reference frame definitions and skeletal size quantifications. The application of force and torque in the laxity tests during surgery is operator dependant, and although these were performed by a single surgeon, this application might have been different over patients. However, intra-surgeon repeatability for these tests was reported to be good [26]. It turned out that four patients (#7, #13, #20 and #32; Table 1) showed joint laxity difference between ADK and CHK not significant during the Lachman, drawer and pivot-shift test, and as such indication for ACL reconstruction is questionable. However, corresponding partial ligament rupture in the ADK was clearly confirmed by surgical examination.

## Conclusions

The present study offers an original contribution to the understanding of laxity and instability at the human knee joint. Measurements were taken with the least possible invasive technique and over a larger number of knee joint laxity tests. The large number of patients analyzed, despite the strict selection criteria, together with normalization for the joint translations also allowed a reliable statistical analysis to be performed, for more robust final findings. The first conclusion is that it is important to measure the antero-posterior and rotational laxity of the uninjured contralateral knee in assessing the laxity of the injured knee. The second is that the Lachman test shows knee laxity better than the AP drawer, and that the pivot-shift test was the only one able to reveal rotational instability.

With these findings, and the support of the current standard surgical navigation systems, the diagnostic evaluation of knee instability can be refined even during surgery. The present original measurements and analyses, here provided for each knee, can contribute to the knowledge of knee joint mechanics, with possible relevant applications in biomedical and clinical research. In particular, the present observations highlight further the importance of a careful and more accurate analysis of the healthy knee in ACL-deficiency, which shall encourage the design of more accurate non-invasive methods for knee laxity quantitative assessment.

### Ethics statement

The data here analyzed are gathered from a series of measurements which are taken usually in the operating room during surgical navigation based ACL reconstructions, and are therefore approved by the institutional scientific review board (Clinique Notre Dame de la Merci, Saint-Raphaël, France). Informed consent was signed by each patient, after explanation of the possible benefits and risks associated to the additional surgical procedures.

## Competing interests

The authors declare that they have no competing interests.

## Authors’ contributions

PI conceived the investigation, performed the surgeries, collected all original data, and drafted the manuscript. CB carried out all the necessary calculations and statistical analyses and contributed significantly into the writing of the manuscript. AL designed this specific study, supervised calculations, and wrote the final manuscript. All authors read and approved the manuscript.
